# Birth and pregnancy numbers decreased during the COVID‐19 pandemic in Japan: A time series analysis with the ARIMA model

**DOI:** 10.1111/jog.16202

**Published:** 2025-01-21

**Authors:** Keiko Yamamoto, Koji Uchiyama, Yoshiko Abe, Nobuko Takaoka, Yasuo Haruyama, Gen Kobashi

**Affiliations:** ^1^ Department of Public Health Dokkyo Medical University, School of Medicine Tochigi Japan; ^2^ Integrated Research Faculty for Advanced Medical Sciences Dokkyo Medical University Tochigi Japan

**Keywords:** birth certificate, COVID‐19, pregnancy, reproductive health, time factors

## Abstract

**Aim:**

The long‐term effects of the COVID‐19 pandemic on birth and pregnancy trends in Japan remain unclear. Although major sporting events are usually followed by an increase in births 9 months later, Japan's fifth wave of COVID‐19 occurred during the Olympics held in Japan during the summer of 2021. In this study, we analyzed how the number of births and pregnancies changed during the COVID‐19 pandemic and large‐scale events in Japan.

**Methods:**

We utilized monthly vital statistical data from birth certificates spanning the years 2010 to 2022. Our analysis followed the identification, estimation, and forecasting stages of autoregressive integrated moving average (ARIMA) modeling. We found the ARIMA (1, 12, 12) model to be adequate for forecasting the monthly number of births.

**Results:**

Comparing actual birth data from 2020 to 2022 with our forecast, we observed a significant decrease in births across all of Japan, urban residential areas, and 13 prefectures—primarily metropolitan regions—in January 2021 and May 2022. We also observed a decrease in pregnancy notifications in May 2020, May 2021, and October 2021. The decrease in births in May 2022 in Japan aligns with the decrease in pregnancy notifications 8 months earlier in October 2021.

**Conclusions:**

Although major sporting events are expected to lead to an increase in the number of births approximately 9 months later, the number of births decreased in May 2022 during the fifth wave of the COVID‐19 pandemic in Japan. These findings suggest that the number of pregnancies and births should be monitored in future pandemics with particular attention to fertility trends.

## INTRODUCTION

On January 30, 2020, following the recommendations of the Emergency Committee, the World Health Organization (WHO) Director General declared the outbreak a public health emergency of international concern.^1^ Many countries imposed restrictive measures such as lockdowns to reduce the spread of the virus in the early stages of the pandemic. These social measures were expected to affect fertility depending on the level of social development in a region, stage of demographic transition, population density, and age distribution.^2^ Analysis of the impact of the COVID‐19 pandemic on birth trends in 38 higher‐income countries until September 2022 revealed that the initial pandemic shock was associated with a fall in the number of births in most countries, with the sharpest drop in January 2021.^3^

During the early days of the COVID‐19 pandemic in April 2020, the Japanese government declared its first state of emergency to slow the spread of infection and reduce the burden on medical care.^4^ Approximately 8–10 months after this declaration, the total number of births in Japan reportedly decreased between December 2020 and February 2021, with metropolitan regions experiencing a greater impact than less urbanized areas.^5^

On May 5, 2023, after the Emergency Committee's assessment of the COVID‐19 pandemic, the WHO Director‐General released a statement stating that COVID‐19 no longer constituted a public health emergency of international concern.^6^ In Japan, countermeasures against COVID‐19, such as self‐quarantine of infected individuals, testing, and public funding of medical expenses, continued until May 7, 2023.^4^

The long‐term effects of these measures on birth and pregnancy trends are unclear. The Olympic and Paralympic Games were held in Japan in 2021 under COVID‐19 countermeasures. Large‐scale events are reportedly associated with an increase in births.^7^ In this study, we analyzed how the number of births and pregnancies changed during the COVID‐19 pandemic and the impact of a large‐scale event in Japan on reproduction.

## METHODS

### Data source

Monthly birth data from birth certificates are released by the Ministry of Health, Labour and Welfare of Japan. In this time series study, we analyzed the number of monthly births from 2010 to 2022 across all of Japan, urban and rural areas, and 47 prefectures.^8^ These statistics pertain to individuals of Japanese nationality.

Based on the definition used for the national census by the Statistics Bureau of Japan, urban areas are regions that encompass all the areas of a city (including the special wards of Tokyo). Rural areas are regions that encompass all the areas of towns and villages.^9^ According to Article 8 of the Local Autonomy Act of Japan, a city is required to have a population of at least 50 000, more than 60% of all households must be located in the central urban area, and more than 60% of the total population must be engaged in commercial, industrial, or other urban occupations. Towns and villages are small‐scale general public entities that do not meet the requirements of a city.

Japan also has a system in place for women to report pregnancies to the municipality, ward, town, or village. To investigate the influence of the COVID‐19 pandemic on couples choosing to postpone pregnancies, the Ministry of Health, Labour and Welfare specifically released the number of monthly pregnancy notifications from January 2018 to October 2021.^10^ Therefore, this time series study also analyzed the monthly number of pregnancy notifications across all of Japan.

Our study adhered to the Strengthening the Reporting of Observational Studies in Epidemiology (STROBE) reporting guidelines. Because we used deidentified publicly available data, there are no ethical concerns regarding data usage.

### Statistical analysis

The auto‐regressive integrated moving average (ARIMA) model combines auto‐regressive (AR) and moving average (MA) components with differencing (Integration). It is widely used for analyzing non‐stationary, autocorrelated time series data, such as excess mortality during a pandemic.^11^, ^12^

In our ARIMA analysis, we utilized the Phillips‐Perron unit root test to assess whether the time series was stationary. After confirming that the original series data on the number of monthly births was non‐stationary, we assessed the autocorrelation with the number of births 12 months prior (using a difference of 12, denoted as *d* = 12). Then, we compared the Akaike Information Criterion (AIC) and Schwartz Bayesian Criterion (SBC) values for different combinations of p and q to find the best‐fit ARIMA model in the Estimation process. In ARIMA (p, d, q) notation, p represents the order of the AR component, d represents the order of differencing, and q represents the order of the MA component.

After finding the best‐fit model, we forecasted the number of births in the forecasting process and compared it with the actual number of births to determine whether there were significant differences across nationwide Japan, urban/rural residences, and the 47 prefectures. Additionally, we examined deviations in the number of monthly pregnancy notifications in Japan using the same model. We compared the forecasted numbers within the upper 95% (U95) and lower 95% (L95) confidence intervals with the actual numbers.

All statistical analyses were performed using SAS Enterprise Guide, version 8.3 (32‐bit).

## RESULTS

### Identification process

**FIGURE 1:**
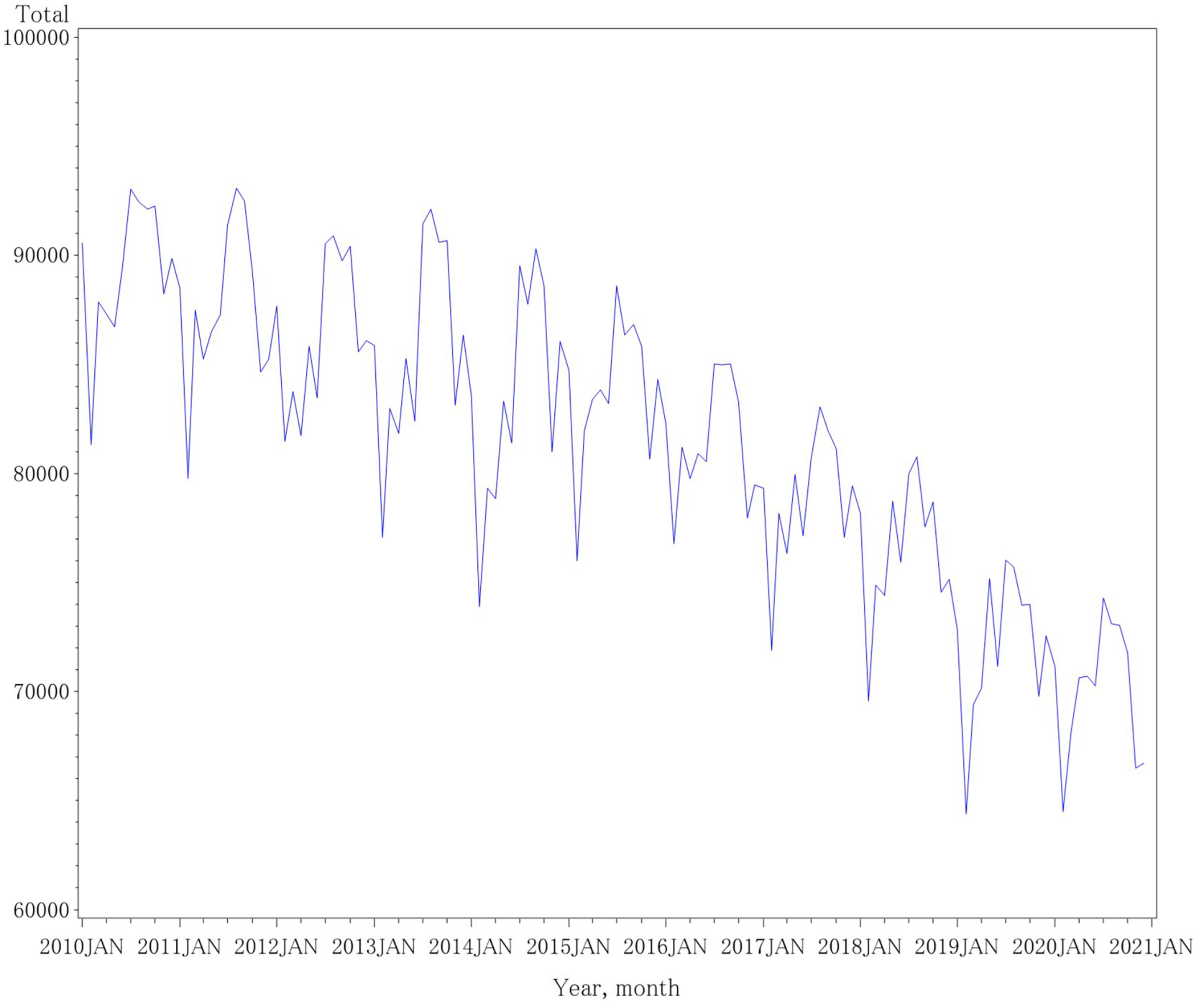
Time series plot of the monthly number of births from January 2010 through December 2020. The monthly number of births from January 2010 through December 2020 in Japan showed a fluctuating decrease and non‐stationarity.

We imported 132 months of birth data from January 2010 through December 2020 into the SAS library to project pandemic birth trends from the pre‐pandemic data. The time series plot of the monthly number of births showed a fluctuating decrease and non‐stationarity (Figure 1).

For the entire country, the mean (standard deviation) of the monthly number of births from January 2010 to December 2020 was 81411.8 (6938.8). The zero‐mean was non‐stationarity at lags 0, 1, or 2 according to the Phillips‐Perron unit root test (all *p* values >0.05; Table S1, Supporting Information). The white noise hypothesis was strongly rejected (*p* < 0.0001; Table S2).

Visual inspection of the autocorrelation function plot also indicated that the number of births was non‐stationary, as the autocorrelation function (ACF) decayed very slowly (Figure S1).

To account for the seasonality of birth, we applied a seasonal differencing of 12 (*d* = 12). After this adjustment, the zero‐mean became stationary at lags 0, 1, and 2 based on the Phillips‐Perron unit root test (all *p* values <0.05; Table S1). The white noise hypothesis remained strongly rejected (Table S2). Visual inspection of the ACF plot also suggested that the number of births became stationary, as the ACF decreased rapidly (Figure S1).

### Estimation process

We compared the AIC and SBC values for different values of p and q. Additionally, we examined the difference of 1 (*d* = 1) with varying p and q. Our findings indicated that the ARIMA (1, 12, 12) had the lowest AIC and SBC values (Table S3). Furthermore, the ARIMA (1, 12, 12) exhibited significant t values in the Conditional Least Squares Estimation for the mean term, the AR parameter, and the MA parameter. Residuals were autocorrelated for all lags (*p* < 0.05; Table S4).

The normality plots of residuals from the ARIMA (1, 12, 12) showed no departure from normality (Figure S2).

Based on these results, we concluded that the ARIMA (1, 12, 12) is an adequate model for forecasting the monthly number of births.

### Forecasting process

#### Change in the number of births at the country level

**FIGURE 2:**
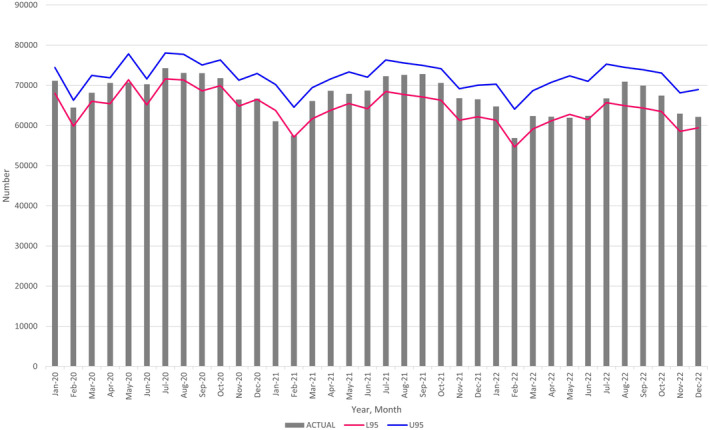
Actual number of monthly births (bar), in all Japan, with 95% prediction intervals of the ARIMA (1, 12, 12) (lines) from January 2020 through December 2022. L95: lower 95% confidence intervals, U95: upper 95% confidence intervals. ARIMA, autoregressive integrated moving average.

By comparing the actual births with the ARIMA (1, 12, 12) forecasted births and their 95% prediction intervals from January 2020 through December 2022, we observed the following trends:

1. The actual number of births fell below the lower 95% prediction intervals, indicating significant decreases in January 2021 and May 2022 (Figure 2 and Table S5).
2. The actual number of births remained below the upper 95% prediction intervals, suggesting no significant increase in births during 2021 and 2022 (Figure 2 and Table S5).

#### Change in the number of births at the regional level

**FIGURE 3:**
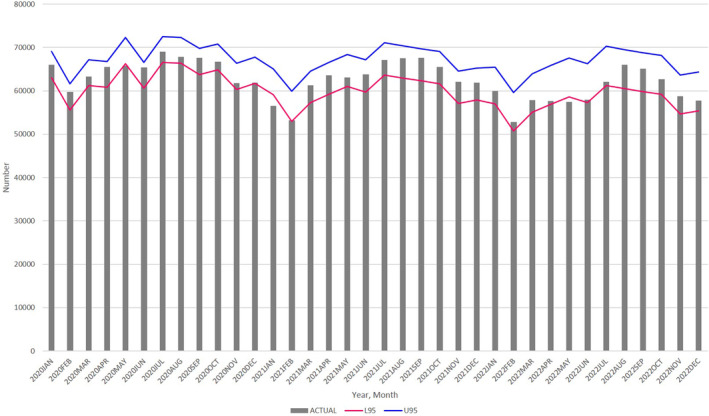
Actual number of monthly births in urban areas (bar), with 95% prediction intervals of the ARIMA (1, 12, 12) (lines) from January 2020 through December 2022. L95: lower 95% confidence intervals, U95: upper 95% confidence intervals. ARIMA, autoregressive integrated moving average.

**FIGURE 4:**
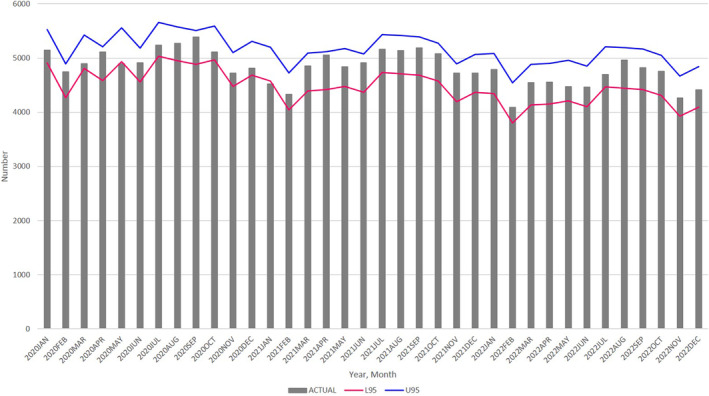
Actual number of monthly births in rural areas (bar), with 95% prediction intervals of the ARIMA (1, 12, 12) (lines) from January 2020 through December 2022. L95: lower 95% confidence intervals, U95: upper 95% confidence intervals. ARIMA, autoregressive integrated moving average.

Considering the impact of the pandemic on metropolitan regions, we examined birth numbers in urban and rural areas using the ARIMA (1, 12, 12) model:

1. In urban areas, we observed significant decreases in January 2021 and May 2022, and no significant increase in births during 2021 and 2022, similar to the nationwide trend (Figure 3, Table S6).
2. In contrast, in rural areas, we observed a decrease in the number of births in January 2021; there was no decrease in May 2022 and no increase during 2021 and 2022 (Figure 4, Table S6).

In Japan, the 47 prefectures were the first‐level administrative divisions for COVID‐19 pandemic countermeasures. At the prefectural level, we identified significant decreases in the number of births in 23 prefectures in January 2021 and in 15 prefectures in May 2022 using the same ARIMA (1, 12, 12) model (Table S7).

Notably, 13 prefectures—Yamagata, Tochigi, Tokyo, Chiba, Kanagawa, Yamanashi, Aichi, Osaka, Hyogo, Nara, Hiroshima, Fukuoka, and Nagasaki—mostly located in metropolitan regions, showed significant decreases in the number of births in both January 2021 and May 2022.

#### Change in the number of pregnancy notifications

The Ministry of Health, Labour and Welfare recommends that pregnant women notify their municipality of their pregnancy within 11 weeks of pregnancy. During fiscal year 2019, 93.5% of pregnant women notified their local municipality of their pregnancies within 11 weeks.^10^

**FIGURE 5:**
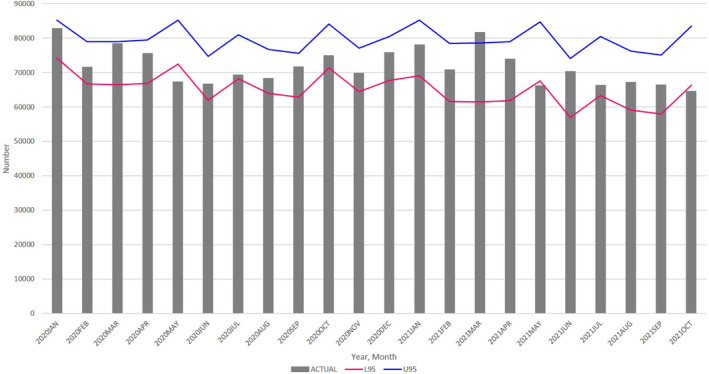
Actual number of pregnancy notifications (bar), with 95% prediction intervals of the ARIMA (1, 12, 12) (lines) from January 2020 through October 2021. L95: lower 95% confidence intervals, U95: upper 95% confidence intervals. ARIMA, autoregressive integrated moving average.

Although the data on monthly pregnancy notifications are available for only a limited period, we observed a significant decrease in May 2020, May 2021, and October 2021, with the actual number of pregnancies falling below the lower 95% prediction intervals. Conversely, in March 2021, the number of pregnancy notifications increased, exceeding the upper 95% prediction intervals (Figure 5, Table S8).

## DISCUSSION

We compared the monthly number of births from 2020 to 2022 and the monthly number of pregnancy notifications from 2020 to October 2021 with the ARIMA forecasts to assess the impact of the COVID‐19 pandemic on conception.

Our analysis demonstrated a significant decrease in the number of births across all of Japan, urban areas, and 13 prefectures (mostly metropolitan regions) in January 2021 and May 2022. Further, the number of pregnancy notifications also decreased in May 2020, May 2021, and October 2021. No excess births were noted during 2021 and 2022.

The decrease in the number of births in January 2021 and in metropolitan regions is consistent with previous research.^5^ The number of pregnancy notifications decreased in May 2020, coinciding with the first wave of the COVID‐19 epidemic and the initial state of emergency. This decrease preceded the birth decline 9 months later, in January 2021, which supports the reliability of our findings.

The present analysis is the first to reveal a decrease in the number of births in Japan in May 2022, 9 months after the Tokyo Olympic and Paralympic Games. A decline in pregnancy notifications occurred earlier in October 2021, coinciding with the fifth wave of the COVID‐19 epidemic and the Tokyo Olympics held from July 23 to August 8, as well as the Tokyo Paralympics held from August 24 to September 5.

A recent systematic review indicated that certain events, including the Super Bowl, the 2009 UEFA Champions League, the 2010 FIFA World Cup, the 2016 UEFA Euros, and the 2019 Rugby World Cup, led to increased birth rates approximately 9 months later.^7^ Despite the significance of the Tokyo Olympics and Paralympics as major sporting events from July to September 2021, the number of births did not follow the expected increasing trend in births approximately 9 months later. In fact, the number of births 9 months later (April to June 2022) decreased in May 2022. In contrast, at 9 months after the 2019 Rugby World Cup held in Japan from September 20, 2019, to November 2, 2019, the number of births did not decrease. These findings suggest that couples may have been hesitant to conceive during the Tokyo Olympics and Paralympics games, possibly due to the ongoing COVID‐19 pandemic. We also found a significant decrease in pregnancy notifications in October 2021, consistent with the decrease in births 8 months later in May 2022.

During the fifth wave of the pandemic, the government declared the fourth state of emergency in Tokyo, which lasted from July 12 to September 30, and decided to hold the Tokyo Olympics and Paralympics games without audiences.^13^ It is noteworthy that a significant decrease in the number of births was observed 9 months after the Tokyo Olympic Games in May 2022, particularly in Tokyo, the main venue for the event.

During the fifth wave of the pandemic in Japan, the number of confirmed COVID‐19 cases and inpatient cases was largest between 2020 and 2021 (Figure S3).^14^ Based on an analysis of excess deaths until June 2021, excess mortality possibly related to COVID‐19 was observed in Japan from April 2021 to June 2021, for the first time since February 2020.^15^ The hospitalization capacity of medical institutions remained limited during the subsequent fifth wave with the largest number of cases. Pregnant women infected with COVID‐19 experiencing only light symptoms stayed at home or in a hotel at that time. On August 17, 2021, a pregnant woman infected with COVID‐19 in Kashiwa, near Tokyo, lost her baby because she was unable to find a hospital for admission.^16^ The news was widely broadcast in Japan, and the Japan Society of Obstetrics and Gynecology issued a notice to pregnant women on August 23, 2021. The notice called on pregnant women infected with COVID‐19 to be vigilant in checking for any abnormal symptoms related to pregnancy in addition to the symptoms associated with COVID‐19.^17^ Additionally, mRNA vaccination gradually expanded to include not only older individuals but also those with underlying diseases and others during the fifth wave of the epidemic. In August 2021, the Japan Society of Obstetrics and Gynecology began recommending that pregnant women receive the mRNA vaccine.^18^ The Japanese government also encouraged local authorities to facilitate early and smooth vaccination for pregnant women and their spouses because vaccination of pregnant women was still in progress.^19^

During the COVID‐19 pandemic, restrictions on deliveries were in place; for example, returning to one's hometown for birth and partner participation during delivery were restricted.^20^ In addition, many facilities performed cesarean sections considering the medical resources available for infection control and the overall condition of pregnant women. A survey of the facilities accounting for 63.3% of deliveries in Japan revealed that more than 80% of these facilities transferred or performed a cesarean section for a pregnant woman infected with COVID‐19 after 37 weeks of gestation.^21^

Considering these circumstances, the fears and anxiety related to COVID‐19 among women in the reproductive age group likely contributed to the decline in fertility intention during the fifth wave of the pandemic. A study in Japan found that approximately one‐fifth of married women who initially intended to have children before the pandemic decided to postpone pregnancy and exhibited deteriorated mental health.^22^ Several studies also suggest higher levels of anxiety and depression in pregnant women during the COVID‐19 pandemic.^23^, ^24^ Our previous analysis of nationwide data provided by the Japan Society of Obstetrics and Gynecology suggested that pregnancy complications and delivery outcomes worsened during the COVID‐19 pandemic.^25^

Couples had lower fertility intentions during the fifth wave of the pandemic, even during the Olympic and Paralympic Games. An unexpected downturn in births in early 2022 was also reported in other countries. Some researchers tried to explain this trend as a pre‐pandemic decline in fertility; birth postponement caused by the resumption of busier work and social lives; earlier pandemic disruptions to dating, partnering, socializing, and marriage; as well as concerns related to vaccination.^3^ Other researchers, however, reported that many uncertainties about the effect of COVID‐19 on fertility and family dynamics remain.^26^

Because fertility intention is influenced by various social conditions such as an individual's psychology, economic situation, and family policies, we should pay attention to healthy reproduction with timely data analysis to handle such unprecedented shocks. Continuing this type of monitoring is also crucial for understanding trends over time. In future pandemics, continued monitoring of the number of pregnancies and births will also be important with close attention to fertility trends.

## LIMITATIONS

Our study has several limitations. The ARIMA model uses pre‐pandemic data to identify deviations. We did not perform a multivariate ARIMA analysis or examine confounding factors, making it difficult to derive causal relationships. Although we utilized the latest vital statics, countermeasures against COVID‐19 were continued until May 2023. Further research is needed to assess the overall impact of the COVID‐19 pandemic on the number of births in Japan. In addition, due to the inability to obtain monthly birth data for the event locations, analyzing the trend of the number of births where the Olympics were held is difficult. Monthly pregnancy notifications are available for only a limited period; therefore, it is difficult to assess the impact of the COVID‐19 pandemic on the pregnancy trend.

## AUTHOR CONTRIBUTIONS

**Keiko Yamamoto:** Conceptualization; data curation; formal analysis; methodology; writing – original draft; writing – review and editing. **Koji Uchiyama:** Conceptualization; formal analysis; methodology; writing – review and editing. **Yoshiko Abe:** Writing – review and editing. **Nobuko Takaoka:** Writing – review and editing. **Yasuo Haruyama:** Writing – review and editing. **Gen Kobashi:** Project administration; supervision; writing – review and editing.

## CONFLICT OF INTEREST STATEMENT

The authors declare no conflicts of interest for this article.

## Supporting information


**Figure S1.** Autocorrelation function (ACF) plots illustrating the effect of differencing on the time series. Without differencing (*d* = 0), the ACF decays very slowly, indicating that the series is non‐stationary. After applying a 12‐month differencing (*d* = 12), the ACF decays rapidly, suggesting that the series has become stationary.

**Figure S2.** Normality plots of the residuals from the ARIMA (1, 12, 12) model, showing no significant departure from normality.

**Figure S3.** During the fifth wave of the COVID‐19 pandemic in Japan, the number of confirmed cases and hospitalizations reached its peak between 2020 and 2021.

**Table S1.** Phillips‐Perron Unit Root Test results examining the stationarity in the identification process. The zero‐mean was non‐stationary at lag 0, 1, or 2 (*p* > 0.05) without differencing (*d* = 0). The zero‐mean became stationary at lag 0, 1, or 2 (*p* < 0.05) with a differencing order of 12 (*d* = 12).
**Table S2**. Autocorrelation check for white noise. The white noise hypothesis was strongly rejected (*p* < 0.0001) both without differencing (*d* = 0) and with a differencing order of 12 (*d* = 12).
**Table S3**. The characteristics of ARIMA models with different parameters. ARIMA (1, 12, 12) had the lowest AIC and SBC. In the components of the ARIMA (p, d, q), p represents the order of the autoregressive component, d represents the order of differencing performed, and q represents the order of the moving average component.
**Table S4**. Parameter estimates and autocorrelation check of residuals for the ARIMA (1, 12, 12). Conditional least squares estimation with the ARIMA (1, 12, 12) had significant *t* values. Autocorrelation check of residuals with the ARIMA (1, 12, 12) indicates significant autocorrelations at all lags (*p* < 0.05).
**Table S5**. Actual and forecasted monthly number of births, with 95% prediction intervals of the ARIMA (1, 12, 12) from January 2020 through December 2022 in Japan.
**Table S6**. Actual and forecasted monthly number of births, with 95% prediction intervals of the ARIMA (1, 12, 12) from January 2020 through December 2022 in urban areas (cities) and rural areas (towns and villages).
**Table S7**. Actual and forecasted monthly number of births, with 95% prediction intervals of the ARIMA (1, 12, 12) in January 2021 and May 2022 in 47 prefectures.
**Table S8**. Actual and forecasted monthly pregnancy notifications, with 95% prediction intervals of the ARIMA (1, 12, 12) from January 2020 through October 2021.

## Data Availability

We analyzed openly available data from Portal Site of Official Statistics of Japan website and Ministry of Health, Labour website available at: https://www.e‐stat.go.jp/en/stat‐search/files?page=1&layout=datalist&toukei=00450011&tstat=000001028897&cycle=7&tclass1=000001053058&tclass2=000001053061&tclass3=000001053064&tclass4val=0, and https://www.mhlw.go.jp/stf/newpage_28374.html.
