# Supplementary figures and images for: Birth and pregnancy numbers decreased during the COVID‐19 pandemic in Japan: A time series analysis with the ARIMA model
Source: J Obstet Gynaecol Res. 2025 Jan 21;51(1):e16202. doi: 10.1111/jog.16202 (PMC11750332; doi:10.1111/jog.16202)

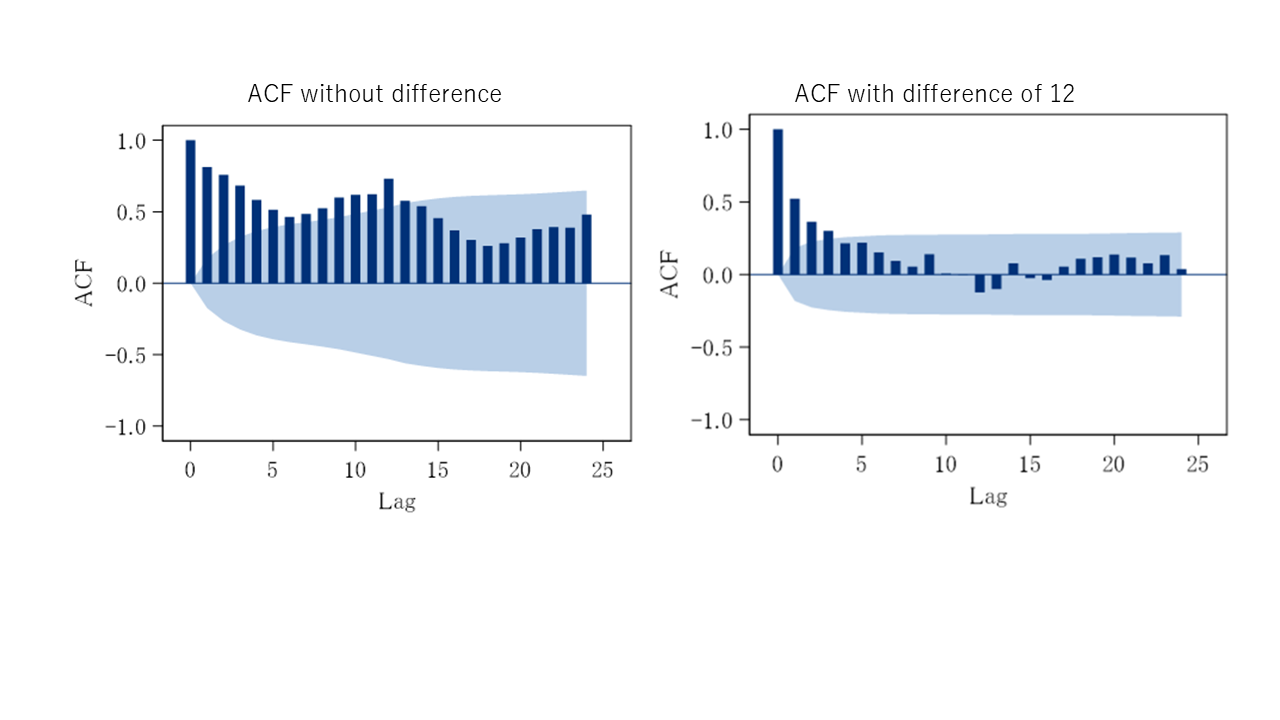

Supplement: Supplementary file 1 — Figure S1. Autocorrelation function (ACF) plots illustrating the effect of differencing on the time series. Without differencing (d = 0), the ACF decays very slowly, indicating that the series is non‐stationary. After applying a 12‐month differencing (d = 12), the ACF decays rapidly, suggesting that the series has become stationary. [file JOG-51-0-s002.tif]

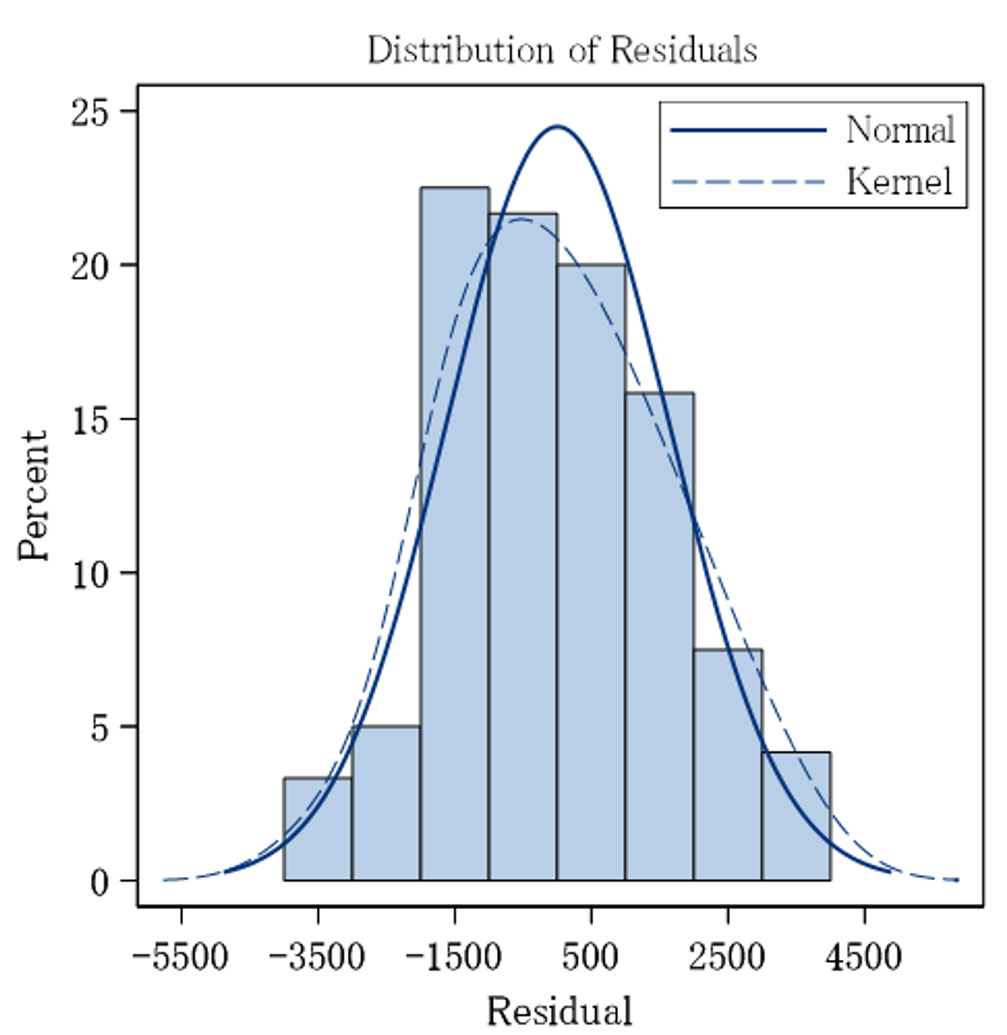

Supplement: Supplementary file 2 — Figure S2. Normality plots of the residuals from the ARIMA (1, 12, 12) model, showing no significant departure from normality. [file JOG-51-0-s003.tif]

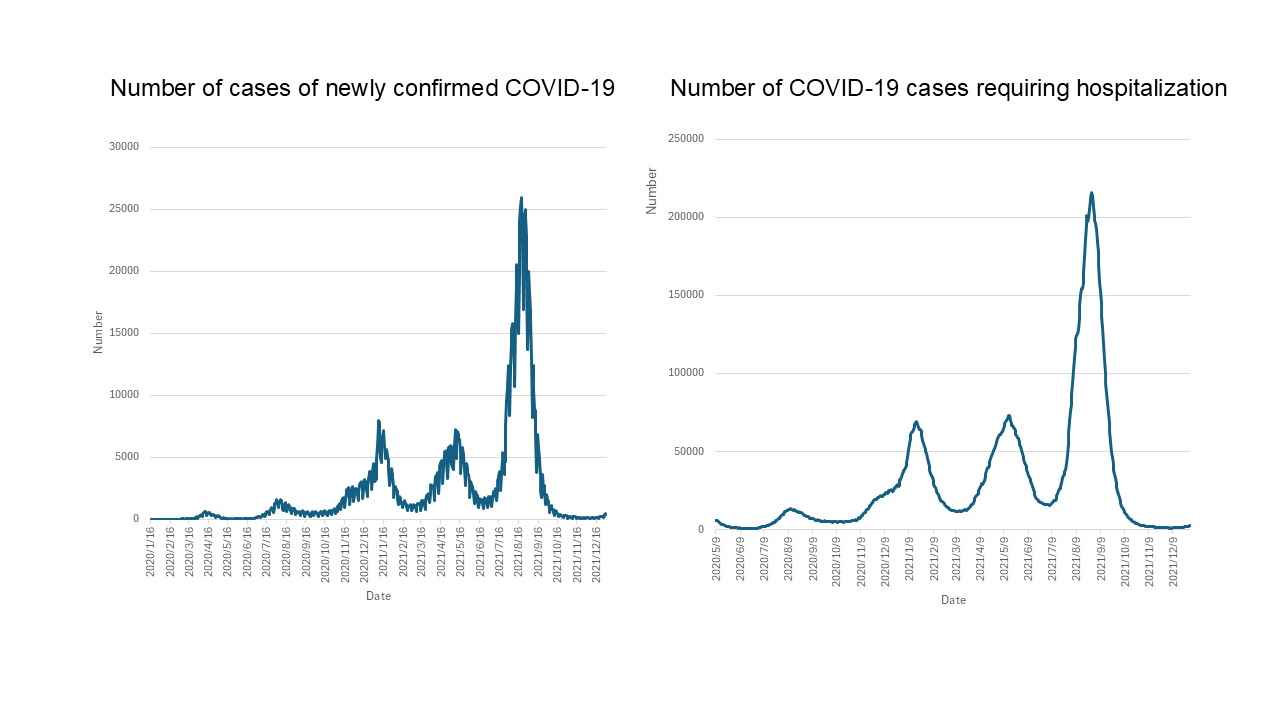

Supplement: Supplementary file 3 — Figure S3. During the fifth wave of the COVID‐19 pandemic in Japan, the number of confirmed cases and hospitalizations reached its peak between 2020 and 2021. [file JOG-51-0-s004.tif]
