# Supplementary material for: Birth and pregnancy numbers decreased during the COVID‐19 pandemic in Japan: A time series analysis with the ARIMA model
Source: J Obstet Gynaecol Res. 2025 Jan 21;51(1):e16202. doi: 10.1111/jog.16202 (PMC11750332; doi:10.1111/jog.16202)
Supplement: Supplementary file 4 — Table S1. Phillips‐Perron Unit Root Test results examining the stationarity in the identification process. The zero‐mean was non‐stationary at lag 0, 1, or 2 (p > 0.05) without differencing (d = 0). The zero‐mean became stationary at lag 0, 1, or 2 (p < 0.05) with a differencing order of 12 (d = 12). Table S2. Autocorrelation check for white noise. The white noise hypothesis was strongly rejected (p < 0.0001) both without differencing (d = 0) and with a differencing order of 12 (d = 12). Table S3. The characteristics of ARIMA models with different parameters. ARIMA (1, 12, 12) had the lowest AIC and SBC. In the components of the ARIMA (p, d, q), p represents the order of the autoregressive component, d represents the order of differencing performed, and q represents the order of the moving average component. Table S4. Parameter estimates and autocorrelation check of residuals for the ARIMA (1, 12, 12). Conditional least squares estimation with the ARIMA (1, 12, 12) had significant t values. Autocorrelation check of residuals with the ARIMA (1, 12, 12) indicates significant autocorrelations at all lags (p < 0.05). Table S5. Actual and forecasted monthly number of births, with 95% prediction intervals of the ARIMA (1, 12, 12) from January 2020 through December 2022 in Japan. Table S6. Actual and forecasted monthly number of births, with 95% prediction intervals of the ARIMA (1, 12, 12) from January 2020 through December 2022 in urban areas (cities) and rural areas (towns and villages). Table S7. Actual and forecasted monthly number of births, with 95% prediction intervals of the ARIMA (1, 12, 12) in January 2021 and May 2022 in 47 prefectures. Table S8. Actual and forecasted monthly pregnancy notifications, with 95% prediction intervals of the ARIMA (1, 12, 12) from January 2020 through October 2021. [file JOG-51-0-s001.docx]

Supplementary Table 1. Phillips-Perron Unit Root Test results examining the stationarity in the identification process. The zero-mean was non-stationary at lag 0, 1, or 2 (P>0.05) without differencing (d=0). The zero-mean became stationary at lag 0, 1, or 2 (P<0.05) with a differencing order of 12 (d=12).

|  | **Phillips-Perron Unit Root Tests** | | | | | | | | | |
| --- | --- | --- | --- | --- | --- | --- | --- | --- | --- | --- |
|  | **d=0** | | | | | **d=12** | | | | |
| **Type** | **Lags** | **Rho** | **Pr < Rho** | **Tau** | **Pr < Tau** | **Lags** | **Rho** | **Pr < Rho** | **Tau** | **Pr < Tau** |
| **Zero Mean** | 0 | -0.44 | 0.58 | -0.78 | **0.3771** | 0 | -26.95 | <0.0001 | -3.72 | **0.0003** |
|  | 1 | -0.38 | 0.60 | -0.85 | **0.3453** | 1 | -22.25 | 0.0006 | -3.39 | **0.0008** |
|  | 2 | -0.37 | 0.60 | -0.88 | **0.3333** | 2 | -20.76 | 0.001 | -3.28 | **0.0012** |
| **Single Mean** | 0 | -20.68 | 0.01 | -3.21 | 0.022 | 0 | -54.73 | 0.0011 | -5.77 | <0.0001 |
|  | 1 | -15.51 | 0.03 | -2.77 | 0.065 | 1 | -51.81 | 0.0011 | -5.65 | <0.0001 |
|  | 2 | -15.58 | 0.03 | -2.78 | 0.064 | 2 | -52.1 | 0.0011 | -5.66 | <0.0001 |
| **Trend** | 0 | -66.34 | 0.0004 | -6.54 | <0.0001 | 0 | -62.64 | 0.0004 | -6.37 | <0.0001 |
|  | 1 | -62.49 | 0.0004 | -6.4 | <0.0001 | 1 | -60.65 | 0.0004 | -6.3 | <0.0001 |
|  | 2 | -66.66 | 0.0004 | -6.55 | <0.0001 | 2 | -61.28 | 0.0004 | -6.32 | <0.0001 |

Supplementary Table 2. Autocorrelation check for white noise. The white noise hypothesis was strongly rejected (p<0.0001) both without differencing (d=0) and with a differencing order of 12 (d=12).

| **Autocorrelation Check for White Noise** | | | | | | | | | |
| --- | --- | --- | --- | --- | --- | --- | --- | --- | --- |
| **d=0** | | | | | | | | | |
| **To Lag** | **Chi-Square** | **DF** | **Pr > ChiSq** | **Autocorrelations** | | | | | |
| **6** | 345.6 | 6 | **<0.0001** | 0.81 | 0.76 | 0.68 | 0.58 | 0.51 | 0.47 |
| **12** | 660.56 | 12 | **<0.0001** | 0.48 | 0.53 | 0.60 | 0.62 | 0.62 | 0.73 |
| **18** | 830.43 | 18 | **<0.0001** | 0.58 | 0.54 | 0.46 | 0.37 | 0.30 | 0.26 |
| **24** | 968.6 | 24 | **<0.0001** | 0.28 | 0.32 | 0.38 | 0.39 | 0.39 | 0.48 |
| **d=12** | | | | | | | | | |
| **To Lag** | **Chi-Square** | **DF** | **Pr > ChiSq** | **Autocorrelations** | | | | | |
| **6** | 76.02 | 6 | **<0.0001** | 0.522 | 0.36 | 0.30 | 0.21 | 0.22 | 0.15 |
| **12** | 82.21 | 12 | **<0.0001** | 0.094 | 0.05 | 0.14 | 0.01 | -0.01 | -0.12 |
| **18** | 86.84 | 18 | **<0.0001** | -0.102 | 0.08 | -0.03 | -0.04 | 0.05 | 0.11 |
| **24** | 97.44 | 24 | **<0.0001** | 0.118 | 0.14 | 0.12 | 0.08 | 0.13 | 0.04 |

Supplementary Table 3. The characteristics of ARIMA models with different parameters. ARIMA (1, 12, 12) had the lowest AIC and SBC. In the components of the ARIMA (p, d, q), p represents the order of the autoregressive component, d represents the order of differencing performed, and q represents the order of the moving average component.

| Models | AIC | SBC |
| --- | --- | --- |
| ARIMA(12,12,12) | 2162.8 | 2171.2 |
| ARIMA(1,12,1) | 2122.7 | 2131.1 |
| ARIMA(12,1,1) | 2355.1 | 2363.7 |
| ARIMA(12,12,1) | 2134.8 | 2143.2 |
| ARIMA(1,1,1) | 2529.6 | 2538.2 |
| ARIMA(1,12,12) | **2120.6** | **2128.9** |

AIC: Akaike information criterion, SBC: Schwartz Bayesian criterion

Supplementary Table 4. Parameter estimates and Autocorrelation Check of Residuals for the ARIMA (1,12,12). Conditional Least Squares Estimation with the ARIMA (1,12,12) had significant t values. Autocorrelation Check of Residuals with the ARIMA (1,12,12) indicates significant autocorrelations at all lags (p<0.05).

| **Conditional Least Squares Estimation** | | | | | | **Autocorrelation Check of Residuals** | | | | | | | | | |
| --- | --- | --- | --- | --- | --- | --- | --- | --- | --- | --- | --- | --- | --- | --- | --- |
| **Parameter** | **Estimate** | **Standard** | **t Value** | **Approx** | **Lag** | **To Lag** | **Chi-Square** | **DF** | **Pr > ChiSq** | **Autocorrelations** | | | | | |
|  |  | **Error** |  | **Pr > \|t\|** |  | **6** | 9.7 | 4 | **0.0459** | -0.118 | 0.087 | 0.161 | -0.048 | 0.143 | 0.084 |
| **MU** | -1930 | 251.6939 | -7.67 | **<0.0001** | 0 | **12** | 22.68 | 10 | **0.012** | 0.038 | -0.038 | 0.261 | -0.098 | 0.115 | 0.065 |
| **MA1,1** | 0.30784 | 0.09712 | 3.17 | **0.0019** | 12 | **18** | 42.73 | 16 | **0.0003** | -0.174 | 0.304 | -0.027 | -0.055 | 0.11 | 0.081 |
| **AR1,1** | 0.57425 | 0.07964 | 7.21 | **<0.0001** | 1 | **24** | 57.39 | 22 | **<0.0001** | 0.027 | 0.124 | 0.084 | -0.066 | 0.229 | -0.132 |

Supplementary Table 5. Actual and forecasted monthly number of births, with 95% prediction intervals of the ARIMA (1,12,12) from January 2020 through December 2022 in Japan.

|  | ACTUAL | FORECAST | L95 | U95 |
| --- | --- | --- | --- | --- |
| Jan-20 | 71146 | 71196.3 | 67975.7 | 74417.0 |
| Feb-20 | 64497 | 63089.4 | 59868.7 | 66310.0 |
| Mar-20 | 68145 | 69268.6 | 66047.9 | 72489.2 |
| Apr-20 | 70623 | 68651.4 | 65430.7 | 71872.1 |
| May-20 | 70724 | 74595.3 | 71374.6 | 77816.0 |
| Jun-20 | 70271 | 68384.3 | 65163.6 | 71605.0 |
| Jul-20 | 74292 | 74820.9 | 71600.2 | 78041.6 |
| Aug-20 | 73095 | 74529.4 | 71308.7 | 77750.1 |
| Sep-20 | 73055 | 71861.1 | 68640.5 | 75081.8 |
| Oct-20 | 71789 | 73121.5 | 69900.8 | 76342.1 |
| Nov-20 | 66489 | 68073.4 | 64852.8 | 71294.1 |
| Dec-20 | 66709 | 69731.4 | 66510.7 | 72952.1 |
| Jan-21 | **61040** | 66971.8 | 63751.1 | 70192.5 |
| Feb-21 | 57533 | 60844.9 | 57131.0 | 64558.9 |
| Mar-21 | 66086 | 65572.0 | 61709.2 | 69434.8 |
| Apr-21 | 68663 | 67716.8 | 63806.1 | 71627.4 |
| May-21 | 67893 | 69425.2 | 65498.8 | 73351.5 |
| Jun-21 | 68694 | 68122.6 | 64191.2 | 72054.1 |
| Jul-21 | 72301 | 72399.4 | 68466.3 | 76332.5 |
| Aug-21 | 72615 | 71628.0 | 67694.3 | 75561.7 |
| Sep-21 | 72844 | 71023.4 | 67089.5 | 74957.2 |
| Oct-21 | 70616 | 70210.8 | 66276.9 | 74144.8 |
| Nov-21 | 66780 | 65248.8 | 61314.8 | 69182.7 |
| Dec-21 | 66557 | 66105.5 | 62171.5 | 70039.5 |
| Jan-22 | 64721 | 65803.6 | 61279.8 | 70327.3 |
| Feb-22 | 56868 | 59352.4 | 54650.3 | 64054.4 |
| Mar-22 | 62352 | 63893.2 | 59133.8 | 68652.5 |
| Apr-22 | 62209 | 65931.0 | 61152.9 | 70709.1 |
| May-22 | **61938** | 67578.0 | 62793.7 | 72362.2 |
| Jun-22 | 62432 | 66240.2 | 61453.9 | 71026.4 |
| Jul-22 | 66757 | 70496.7 | 65709.7 | 75283.6 |
| Aug-22 | 70950 | 69713.7 | 64926.5 | 74500.9 |
| Sep-22 | 69953 | 69102.3 | 64315.1 | 73889.6 |
| Oct-22 | 67449 | 68286.0 | 63498.7 | 73073.2 |
| Nov-22 | 62967 | 63321.7 | 58534.4 | 68109.0 |
| Dec-22 | 62163 | 64177.2 | 59389.9 | 68964.5 |

L95: lower 95% confidence intervals, U95: upper 95% confidence intervals

Note: Yellow highlighting indicates that the actual number of births fell below the lower 95% prediction intervals, revealing significant decreases in January 2021 and May 2022.

Supplementary Table 6. Actual and forecasted monthly number of births, with 95% prediction intervals of the ARIMA (1,12,12) from January 2020 through December 2022 in urban areas (cities) and rural areas (towns and villages).

|  | urban area (cities) | | | | rural area (towns and villages) | | | |
| --- | --- | --- | --- | --- | --- | --- | --- | --- |
|  | ACTUAL | FORECAST | L95 | U95 | ACTUAL | FORECAST | L95 | U95 |
| 2020JAN | 65993 | 66029.0 | 63024.8 | 69033.3 | 5149 | 5217.8 | 4906.9 | 5528.8 |
| 2020FEB | 59751 | 58600.0 | 55595.8 | 61604.2 | 4743 | 4578.0 | 4267.1 | 4889.0 |
| 2020MAR | 63245 | 64173.7 | 61169.5 | 67177.9 | 4899 | 5118.3 | 4807.4 | 5429.3 |
| 2020APR | 65509 | 63768.2 | 60763.9 | 66772.4 | 5111 | 4895.1 | 4584.2 | 5206.1 |
| 2020MAY | 65829 | 69305.7 | 66301.5 | 72310.0 | 4894 | 5245.1 | 4934.2 | 5556.1 |
| 2020JUN | 65351 | 63590.0 | 60585.8 | 66594.2 | 4916 | 4869.0 | 4558.0 | 5179.9 |
| 2020JUL | 69055 | 69526.8 | 66522.6 | 72531.0 | 5236 | 5344.6 | 5033.7 | 5655.6 |
| 2020AUG | 67823 | 69346.0 | 66341.8 | 72350.2 | 5269 | 5264.1 | 4953.2 | 5575.1 |
| 2020SEP | 67665 | 66755.8 | 63751.6 | 69760.0 | 5388 | 5195.3 | 4884.4 | 5506.3 |
| 2020OCT | 66670 | 67833.3 | 64829.1 | 70837.5 | 5117 | 5280.2 | 4969.2 | 5591.1 |
| 2020NOV | 61767 | 63318.3 | 60314.1 | 66322.5 | 4721 | 4785.9 | 4475.0 | 5096.9 |
| 2020DEC | 61895 | 64755.8 | 61751.5 | 67760.0 | 4812 | 4996.8 | 4685.8 | 5307.7 |
| 2021JAN | 56517 | 62084.6 | 59080.4 | 65088.8 | 4522 | 4890.7 | 4579.8 | 5201.7 |
| 2021FEB | 53200 | 56393.7 | 52909.6 | 59877.7 | 4332 | 4385.9 | 4043.2 | 4728.7 |
| 2021MAR | 61226 | 60881.7 | 57246.7 | 64516.7 | 4858 | 4741.4 | 4392.1 | 5090.6 |
| 2021APR | 63604 | 62869.0 | 59183.4 | 66554.6 | 5058 | 4768.2 | 4417.5 | 5118.8 |
| 2021MAY | 63056 | 64708.4 | 61005.5 | 68411.3 | 4837 | 4827.1 | 4476.2 | 5178.0 |
| 2021JUN | 63776 | 63434.5 | 59725.6 | 67143.3 | 4917 | 4723.0 | 4372.0 | 5074.0 |
| 2021JUL | 67137 | 67390.7 | 63679.8 | 71101.6 | 5163 | 5083.8 | 4732.8 | 5434.8 |
| 2021AUG | 67471 | 66645.7 | 62934.1 | 70357.3 | 5142 | 5063.4 | 4712.3 | 5414.4 |
| 2021SEP | 67659 | 65989.7 | 62277.9 | 69701.6 | 5184 | 5037.7 | 4686.7 | 5388.7 |
| 2021OCT | 65532 | 65370.3 | 61658.4 | 69082.3 | 5083 | 4926.7 | 4575.7 | 5277.7 |
| 2021NOV | 62060 | 60813.0 | 57101.0 | 64525.0 | 4720 | 4542.2 | 4191.2 | 4893.2 |
| 2021DEC | 61837 | 61566.1 | 57854.2 | 65278.1 | 4720 | 4715.0 | 4364.0 | 5066.0 |
| 2022JAN | 59933 | 61200.8 | 56964.7 | 65436.8 | 4788 | 4713.8 | 4345.2 | 5082.4 |
| 2022FEB | 52778 | 55183.8 | 50781.5 | 59586.2 | 4090 | 4171.9 | 3799.6 | 4544.2 |
| 2022MAR | 57806 | 59480.4 | 55022.1 | 63938.7 | 4546 | 4510.1 | 4137.0 | 4883.2 |
| 2022APR | 57652 | 61355.3 | 56877.8 | 65832.7 | 4557 | 4528.9 | 4155.7 | 4902.2 |
| 2022MAY | 57467 | 63128.6 | 58644.6 | 67612.7 | 4471 | 4584.2 | 4210.9 | 4957.4 |
| 2022JUN | 57969 | 61815.9 | 57329.6 | 66302.2 | 4463 | 4478.4 | 4105.1 | 4851.6 |
| 2022JUL | 62057 | 65749.3 | 61262.2 | 70236.4 | 4698 | 4838.4 | 4465.1 | 5211.7 |
| 2022AUG | 65983 | 64990.9 | 60503.6 | 69478.3 | 4966 | 4817.5 | 4444.2 | 5190.8 |
| 2022SEP | 65135 | 64327.1 | 59839.6 | 68814.5 | 4818 | 4791.7 | 4418.4 | 5165.0 |
| 2022OCT | 62695 | 63703.1 | 59215.6 | 68190.5 | 4754 | 4680.6 | 4307.3 | 5053.9 |
| 2022NOV | 58703 | 59143.0 | 54655.5 | 63630.5 | 4262 | 4296.1 | 3922.8 | 4669.4 |
| 2022DEC | 57745 | 59894.6 | 55407.1 | 64382.1 | 4414 | 4468.9 | 4095.6 | 4842.2 |

L95: lower 95% confidence intervals, U95: upper 95% confidence intervals

Note: Yellow highlighting indicates that the actual number of births fell below the lower 95% prediction intervals, revealing significant decreases.

Supplementary Table 7. Actual and forecasted monthly number of births, with 95% prediction intervals of the ARIMA (1,12,12) in January 2021 and May 2022 in 47 prefectures.

| Prefecture | Jan-21 | | | | May-22 | | | |
| --- | --- | --- | --- | --- | --- | --- | --- | --- |
|  | ACTUAL | FORECAST | L95 | U95 | ACTUAL | FORECAST | L95 | U95 |
| Hokkaido | 2250 | 2430.0 | 2242.9 | 2617.1 | 2192 | 2397.3 | 2186.6 | 2608.0 |
| Aomori | 484 | 534.4 | 458.9 | 609.9 | 501 | 570.8 | 486.9 | 654.8 |
| Iwate | 513 | 565.7 | 486.2 | 645.1 | 519 | 551.6 | 467.2 | 636.0 |
| Miyagi | 1081 | 1190.2 | 1041.8 | 1338.5 | 1009 | 1220.8 | 1057.1 | 1384.5 |
| Akita | 345 | 375.8 | 320.5 | 431.1 | 335 | 355.5 | 297.1 | 414.0 |
| Yamagata | 434 | 504.2 | 446.5 | 562.0 | 424 | 513.6 | 451.9 | 575.2 |
| Fukushima | 812 | 881.4 | 771.5 | 991.4 | 829 | 901.9 | 760.3 | 1043.5 |
| Ibaraki | 1256 | 1445.2 | 1318.2 | 1572.2 | 1267 | 1398.1 | 1258.2 | 1538.1 |
| Tochigi | 864 | 1007.1 | 896.2 | 1118.1 | 859 | 990.9 | 867.8 | 1114.0 |
| Gunma | 849 | 961.7 | 854.7 | 1068.6 | 842 | 894.5 | 777.0 | 1012.0 |
| Saitama | 3280 | 3763.6 | 3280.0 | 3763.6 | 3425 | 3872.8 | 3548.2 | 4197.5 |
| Chiba | 2844 | 3206.3 | 2996.1 | 3416.5 | 2953 | 3265.6 | 3038.8 | 3492.4 |
| Tokyo | 6656 | 7535.2 | 7015.1 | 8055.4 | 7302 | 8526.1 | 7671.3 | 9381.0 |
| Kanagawa | 4287 | 4884.6 | 4565.1 | 5204.1 | 4526 | 4988.1 | 4587.3 | 5388.9 |
| Niigata | 1004 | 1067.3 | 962.6 | 1172.1 | 967 | 1055.5 | 938.7 | 1172.2 |
| Toyama | 428 | 517.2 | 457.9 | 576.6 | 538 | 514.7 | 453.7 | 575.7 |
| Ishikawa | 560 | 620.2 | 549.6 | 690.8 | 555 | 631.8 | 548.7 | 715.0 |
| Fukui | 393 | 435.0 | 381.7 | 488.3 | 401 | 434.5 | 378.5 | 490.5 |
| Yamanashi | 367 | 431.0 | 371.7 | 490.4 | 350 | 413.5 | 351.5 | 475.4 |
| Nagano | 923 | 1050.3 | 954.5 | 1146.0 | 1006 | 1027.8 | 922.0 | 1133.6 |
| Gifu | 960 | 998.7 | 888.0 | 1109.3 | 913 | 958.6 | 837.0 | 1080.2 |
| Shizuoka | 1590 | 1803.0 | 1643.6 | 1962.5 | 1665 | 1786.2 | 1605.8 | 1966.5 |
| Aichi | 4201 | 4556.2 | 4271.9 | 4840.5 | 4091 | 4507.2 | 4164.0 | 4850.4 |
| Mie | 817 | 913.1 | 826.7 | 999.5 | 807 | 896.0 | 802.3 | 989.8 |
| Shiga | 737 | 865.0 | 779.5 | 950.4 | 822 | 815.6 | 717.7 | 913.5 |
| Kyoto | 1136 | 1357.1 | 1245.1 | 1469.1 | 1266 | 1339.1 | 1214.0 | 1464.2 |
| Osaka | 4450 | 5041.0 | 4752.5 | 5329.5 | 4552 | 5067.2 | 4746.0 | 5388.3 |
| Hyogo | 2656 | 2939.8 | 2750.7 | 3128.9 | 2781 | 3009.0 | 2784.9 | 3233.2 |
| Nara | 578 | 682.4 | 601.1 | 763.6 | 564 | 654.5 | 565.6 | 743.3 |
| Wakayama | 421 | 469.7 | 410.2 | 529.3 | 435 | 462.5 | 396.2 | 528.8 |
| Tottori | 312 | 307.9 | 260.0 | 355.8 | 299 | 326.2 | 273.4 | 379.0 |
| Shimane | 354 | 369.8 | 313.7 | 425.9 | 335 | 361.4 | 299.5 | 423.4 |
| Okayama | 1027 | 1113.9 | 1019.5 | 1208.3 | 984 | 1081.1 | 982.3 | 1179.8 |
| Hiroshima | 1469 | 1647.9 | 1512.9 | 1782.8 | 1412 | 1573.1 | 1424.9 | 1721.3 |
| Yamaguchi | 630 | 705.1 | 629.3 | 781.0 | 623 | 645.5 | 566.9 | 724.1 |
| Tokushima | 350 | 373.3 | 319.8 | 426.7 | 308 | 347.0 | 287.1 | 407.0 |
| Kagawa | 479 | 514.1 | 446.9 | 581.4 | 430 | 492.0 | 420.1 | 563.8 |
| Ehime | 633 | 696.9 | 618.8 | 775.1 | 626 | 649.5 | 565.8 | 733.2 |
| Kouchi | 330 | 347.1 | 296.0 | 398.1 | 309 | 330.1 | 277.2 | 382.9 |
| Fukuoka | 2846 | 3211.3 | 3030.4 | 3392.3 | 2841 | 3224.3 | 2982.3 | 3466.2 |
| Saga | 458 | 519.6 | 462.6 | 576.6 | 435 | 476.1 | 416.6 | 535.7 |
| Nagasaki | 698 | 791.2 | 708.5 | 873.9 | 639 | 736.7 | 647.8 | 825.6 |
| Kumamoto | 1020 | 1091.7 | 983.5 | 1199.8 | 988 | 1054.9 | 937.0 | 1172.9 |
| Oita | 574 | 629.7 | 554.8 | 704.7 | 524 | 592.1 | 508.4 | 675.9 |
| Miyazaki | 622 | 640.5 | 579.2 | 701.8 | 550 | 601.2 | 535.5 | 666.8 |
| Kagoshima | 873 | 982.3 | 884.7 | 1079.9 | 819 | 913.8 | 803.1 | 1024.6 |
| Okinawa | 1188 | 1229.3 | 1120.5 | 1338.1 | 1120 | 1247.2 | 1118.8 | 1375.6 |

L95: lower 95% confidence intervals,U95: upper 95% confidence intervals

Note: Yellow highlighting indicates that the accrual number of births fell below the lower 95% prediction intervals, revealing significant decreases in January 2021 (23 prefectures) and May 2022 (15 prefectures).

Supplementary Table 8. Actual and forecasted monthly pregnancy notifications, with 95% prediction intervals of the ARIMA (1,12,12) from January 2020 through October 2021.

|  | ACTUAL | FORECAST | L95 | U95 |
| --- | --- | --- | --- | --- |
| 2020JAN | 82809 | 79674.7 | 74177.4 | 85172.0 |
| 2020FEB | 71475 | 72853.2 | 66699.7 | 79006.8 |
| 2020MAR | 78329 | 72698.5 | 66389.7 | 79007.2 |
| 2020APR | 75535 | 73160.2 | 66812.8 | 79507.7 |
| 2020MAY | **67256** | 78839.4 | 72482.2 | 85196.5 |
| 2020JUN | 66680 | 68286.8 | 61927.2 | 74646.4 |
| 2020JUL | 69271 | 74598.6 | 68238.4 | 80958.9 |
| 2020AUG | 68291 | 70335.6 | 63975.2 | 76695.9 |
| 2020SEP | 71615 | 69230.1 | 62869.7 | 75590.5 |
| 2020OCT | 74839 | 77673.6 | 71313.2 | 84034.1 |
| 2020NOV | 69751 | 70772.3 | 64411.8 | 77132.7 |
| 2020DEC | 75747 | 74069.2 | 67708.7 | 80429.6 |
| 2021JAN | 78036 | 77096.1 | 69037.0 | 85155.1 |
| 2021FEB | 70730 | 70025.5 | 61590.8 | 78460.3 |
| 2021MAR | **81677** | 69996.1 | 61468.9 | 78523.2 |
| 2021APR | 73916 | 70394.8 | 61844.4 | 78945.2 |
| 2021MAY | **66192** | 76105.6 | 67549.4 | 84661.9 |
| 2021JUN | 70265 | 65537.1 | 56979.4 | 74094.9 |
| 2021JUL | 66259 | 71857.0 | 63298.9 | 80415.1 |
| 2021AUG | 67158 | 67589.9 | 59031.7 | 76148.0 |
| 2021SEP | 66350 | 66486.4 | 57928.2 | 75044.7 |
| 2021OCT | **64551** | 74929.0 | 66370.7 | 83487.2 |

L95: lower 95% confidence intervals,U95: upper 95% confidence intervals

Note: Yellow highlighting indicates that the accrual number of pregnancy notifications fell below the lower 95% prediction intervals, revealing significant decreases in May 2020, May 2021, and October 2021. Yellow highlighting also shows that the accrual number of pregnancy notifications exceeded the upper 95% prediction intervals, indicating a significant increase in March 2021.
